# Definitions of Frailty in Qualitative Research: A Qualitative Systematic Review

**DOI:** 10.1155/2021/6285058

**Published:** 2021-06-02

**Authors:** Deborah A. Lekan, Susan K. Collins, Audai A. Hayajneh

**Affiliations:** ^1^University of North Carolina at Greensboro, School of Nursing, Nursing and Instructional Building, Greensboro, NC 27402, USA; ^2^Jordan University of Science and Technology, Faculty of Nursing, Ar Ramtha, Jordan

## Abstract

The purpose of this qualitative systematic review was to examine how frailty was conceptually and operationally defined for participant inclusion in qualitative research focused on the lived experience of frailty in community-living frail older adults. Search of six electronic databases, 1994–2019, yielded 25 studies. Data collection involved extracting the definition of frailty from the study aim, background, literature review, methods, and sampling strategy in each research study. Quality appraisal indicated that 13 studies (52%) demonstrated potential researcher bias based on insufficient information about participant recruitment, sampling, and relationship between the researcher and participant. Content analysis and concept mapping were applied for data synthesis. Although frailty was generally defined as a multidimensional, biopsychosocial construct with loss of resilience and vulnerability to adverse outcomes, most studies defined the study population based on older age and physical impairments derived from subjective assessment by the researcher, a healthcare professional, or a family member. However, 13 studies (52%) used objective or performance-based quantitative measures to classify participant frailty. There was no consistency across studies in standardized measures or objective assessment of frailty. Synthesis of the findings yielded four themes: Time, Vulnerability, Loss, and Relationships. The predominance of older age and physical limitations as defining characteristics of frailty raises questions about whether participants were frail, since many older adults at advanced age and with physical limitations are not frail. Lack of clear criteria to classify frailty and reliance on subjective assessment introduces the risk for bias, threatens the validity and interpretation of findings, and hinders transferability of findings to other contexts. Clear frailty inclusion and exclusion criteria and a standardized approach in the reporting of how frailty is conceptually and operationally defined in study abstracts and the methodology used is necessary to facilitate dissemination and development of metasynthesis studies that aggregate qualitative research findings that can be used to inform future research and applications in clinical practice to improve healthcare.

## 1. Introduction

The rapid growth in the aging population globally in terms of both number and increasing longevity has drawn attention to the needs of older persons, especially those who are frail [1]. Frailty has been characterized as nonresilient or accelerated aging and the cumulative effect of detrimental physiologic changes and failed integrative responses [2]. Although frailty increases with age, it differs from normal aging and represents the cumulative effect of aging processes, morbidity, and psychosocial, behavioral, and environmental factors on health and well-being [3]. Frailty is a clinical condition that has been used to describe a person who is very old and who may appear thin, weak, fragile, and feeble. However, frailty is also recognized as a state of vulnerability that may not be visibly apparent but is associated with reduced resilience and poor response to and recovery from acute illness and other stressors. Although some hold the opinion, “I know it when I see it,” it is also acknowledged that not everyone sees the same thing when it comes to frailty [4]. Managing frailty is recognized as an important component in tailoring healthcare for frail older adults.

### 1.1. Literature Review

A growing body of research has contributed new understanding about frailty; however, the views of older adults who are frail may not be included in this scientific work. Despite a proliferation of frailty frameworks and some agreement on aspects of physical frailty, the precise defining characteristics and clinical indicators for frailty and how to measure them in diverse care contexts (e.g., community, nursing home, and acute care hospital) are still evolving [2, 4]. Much of the frailty literature follows a biomedical paradigm focused on pathophysiological etiology, physical function, and phenotypic features of frailty [2, 5]. Despite a large variety of frameworks on ways to assess frailty, the value of frailty assessment is undisputed because of the serious nature of this condition and its consequences. A previous consensus conference on frailty endorsed the usefulness of defining frailty in clinical settings and the need for a clear conceptual framework, indicating that frailty was a clinical syndrome associated with increased vulnerability and potential for preventing, delaying, or reversing frailty with interventions [5]. There is also recognition that there is no ideal frailty assessment tool; the selection of a tool is based on the population characteristics, available data, clinical context, and purpose of the assessment [4, 6, 7].

What is frailty and how it should be assessed have not been informed by qualitative literature in meaningful ways that capture the voice of older adults who are frail [2, 8, 9]. The development of quantitative methodologies for assessment of a phenomenon such as frailty is ideally preceded by qualitative research to explore concepts and correlates. The validity of concepts used in quantitative research can be strengthened by being grounded in real-life experiences and verified in first person encounters through interviews and focus groups derived from qualitative research [10, 11]. Current operational definitions of frailty in the empirical literature typically focus on physical aspects of frailty and overlook psychosocial aspects and other factors that may be more meaningful to frail older adults [5, 8].

An understanding of how frailty is defined in qualitative research studies could inform the dialogue and development of frailty assessment frameworks that can be applied in research investigations and in clinical practice. Qualitative research provides insights that are difficult to produce with quantitative measures by providing detailed descriptions of phenomenon of concern such as frailty in real-life contexts. Synthesized qualitative research findings in the format of a metasynthesis provide new knowledge about a topic with greater influence compared to singular studies. An initial scan of the qualitative research literature on the lived experience of frailty in community-living frail, older adults yielded a very small number of articles and no metasynthesis studies. Following screening of a set of articles, we noted that many lacked a clear conceptual and operational definition of frailty, and inclusion criteria for sample selection were ambiguous and often based on older age or the presence of physical impairments.

By design, qualitative research can be less structured and more open-ended and flexible compared to quantitative research; indeed, a recent systematic review of definitions of qualitative research found that there is no consensus about specific qualitative methods or data analysis guidelines [12]. Considering this and the insights from our initial scan of the literature raised concerns about potential methodological issues related to researcher perspectives about frailty that may contribute to bias in ways that would impact the validity, interpretation, and transferability of study findings. Methodological deficits would also prevent inclusion of many articles in qualitative metasynthesis studies. The purpose of this qualitative systematic review was to perform an in-depth analysis of conceptual and operational definitions of frailty applied in participant sampling and inclusion criteria in qualitative research studies that focused on the lived experience of frailty in community-living, frail older adults.

## 2. Method

### 2.1. Study Design

This study used a qualitative systematic review [13, 14]. This review uses a process which summarizes primary qualitative research studies on a topic derived from a systematic and rigorous literature search [13]. The research evidence is integrated, compared, and synthesized into a holistic interpretation informed by existing theory and research to more fully comprehend the multiple levels of understanding around a topic [11, 14]. Methods for systematic reviews of quantitative research are well established; however, the first publication of a systematic review of qualitative research was not until 2013 [13]. The qualitative systematic review could be an important contribution to the science of frailty since the findings can serve as a foundation to develop new insights and propose strategies for future knowledge development [15]. Sandelowski and Barroso's [10] methodology was employed to synthesize primary qualitative research study findings into a holistic interpretation about the researchers' definitions of frailty that was used for participant inclusion criteria in qualitative research on the lived experience of frailty among frail older adults living in the community.

### 2.2. Literature Search Strategy

A systematic search of six databases was conducted: Cumulative Index to Nursing and Allied Health Literature (CINAHL), MEDLINE (PubMed), ProQuest, PsycINFO, Scopus, and Sociological Abstracts, from their inception through June 2019. The appropriate medical subject headings (MeSH), search terms, and keywords for each database were applied and included the following: “frail elderly” and “qualitative research” (PubMed); “frail” and “middle aged 45–64 years,” “aged 65+ years,” “aged, 80 and over” (CINAHL); “health impairment,” “aging,” “middle age (40–64 years),” “aged (65 years & older),” “very old (85 years & older),” “qualitative” (PsycINFO); and “aging,” “elder,” “frail,” “gerontology,” “geriatric,” “senior,” and “qualitative methods” (Sociological Abstracts). The search was limited to human subjects and English language. Additional citations were located through hand search of reference lists. Citations were excluded if there was no evidence of the older adult's perspective on frailty. Unpublished papers (e.g., abstracts, dissertations, and conference proceedings) were excluded. Study populations that focused on older adults in acute care hospitals or long-term care settings (e.g., assisted living, nursing homes, and rehabilitation facilities) were excluded since the experience of frailty in these contexts would be different. Studies that addressed end-of-life, advance directives, and failure-to-thrive were excluded because these issues are associated with the latest stages of frailty and not the focus of this review.

**Figure 1:**
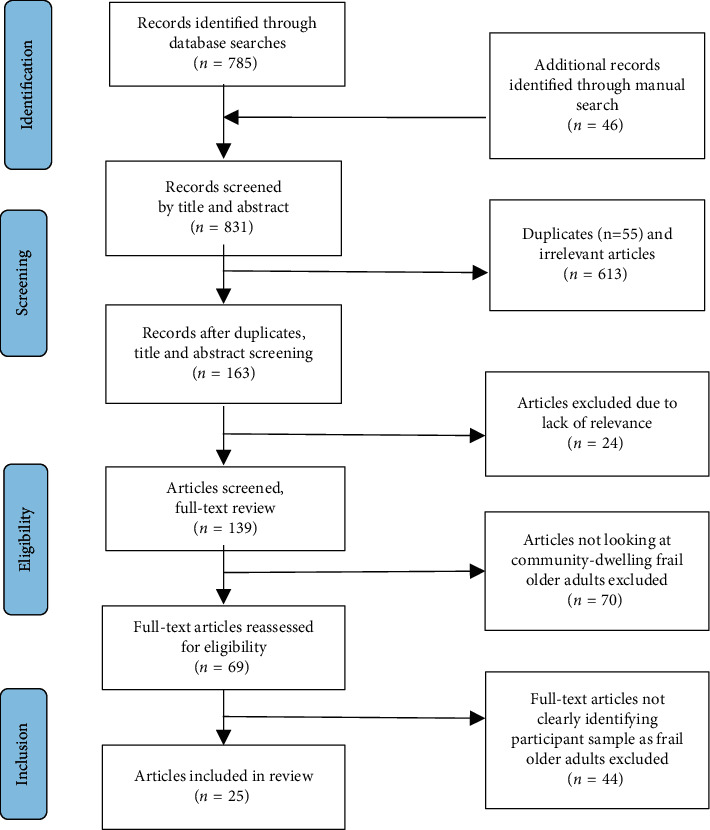
Search strategy flow diagram.

The search yielded 784 citations; an additional 46 articles were identified manually from author searches and reference lists. Two reviewers independently screened titles and abstracts and rated each citation as meeting screening criteria. Full-text reviews of 138 articles were conducted with exclusion of 70 articles that were not focused on community-dwelling frail older adults. A total of 69 articles underwent a second round of full-text review, with exclusions applied due to the study sample not clearly identified as frail, and 44 were excluded. The final sample included 25 articles. Fourteen of these articles were authored by seven researcher groups (two different studies for each group) in which different research questions were examined in the same participant sample [16–29]. The search strategy is shown in Figure 1.

### 2.3. Data Abstraction

A first review of the article was conducted by one coauthor. Articles were read in detail with a focus on the study aim or purpose, background and literature review, and methods. Data abstraction included the following: authors, year of publication, first author professional discipline, country of origin of the research, study aim, conceptual and operational definition of frailty, participant recruitment and characteristics, study setting, and ethical considerations. Statements used to define frailty were extracted and entered verbatim into individual data forms. The conceptual definition of frailty referred to how the term frailty was used by the researcher to describe the construct and guide the sample selection. The operational definition of frailty identified how frailty was measured and classified for inclusion in the study. Specific attention was directed to the methods section (e.g., recruitment, sampling, and inclusion and exclusion criteria) of the article. When an explicit definition of frailty could not be determined, descriptive terminology for frailty was identified. Care was taken not to confound the researcher's definition of frailty for the purpose of the study with the study findings and interpretation. Frailty statements and terminology were transferred into a Microsoft Excel^©^ spreadsheet to facilitate grouping into topical areas. A second review of the article was performed by another coauthor to verify the data abstraction in the data forms and the spreadsheet, making any additions or corrections needed.

### 2.4. Quality Appraisal

Each article underwent quality appraisal with a focus on the research question, methods, and ethics using the Critical Appraisal Skills Programme (CASP) Qualitative Studies Checklist [30]. There are varying opinions about how quality should be assessed, who should assess quality, and whether quality should be assessed in qualitative research at all given the nature of this research genre [31]. It is also proposed that qualitative research is not generalizable and is specific to a certain context and participant group [31]. In accordance with these and other opinions, we did not exclude any articles based on quality [10], especially since this review was prompted by methodological concerns about how researchers classified participants as frail for qualitative studies on the lived experience of frailty. This qualitative systematic review was undertaken to provide a synthesis of articles that met our inclusion criteria because of its potential to inform the design of future qualitative research on frailty.

### 2.5. Data Analysis: Content Analysis and Concept Mapping

Content analysis [32] was undertaken to identify insights and concepts from statements extracted from each article and coded in the spreadsheets. Coding is the process in which data are reduced into manageable units and categorized for meaningful use. Concept mapping was employed to facilitate reflection and understanding about frailty and to graphically organize and represent key concepts in the data aggregation and reduction process [33]. Concept maps are graphical tools for organizing and representing concepts to facilitate identification of similarities, differences, and patterns in the data [33]. Colored-coded sticky notes with concepts/statements for the conceptual and operational definitions of frailty from the spreadsheet were clustered on a white board for the initial graphical organization of the concepts; this visual display was then replicated in a Microsoft PowerPoint^©^ format.

For the synthesis, the color-coded concept groupings were compared, contrasted, and organized into categories. Finally, by collapsing and expanding categories, topical areas emerged, and themes were formed. An iterative process of analysis of concepts produced further refinements leading to classification of data into categories and themes. To ensure validity, the articles were re-reviewed to ensure accurate representation of the concepts, categories, and themes. Regular meetings among our team fostered discussion and interrogation of the data and development of consensus on the categories and themes. The concept map, date-stamped spreadsheets, and meeting notes were maintained for an audit trail and revisited as needed to follow decision-making.

## 3. Results

### 3.1. Systematic Review Findings

The 25 articles in Table 1 represented various disciplines including nursing (11), medicine and neuroscience (3), sociology and social work (6), occupational therapy (2), dentistry (2), and radiology (1). There was a diverse representation by country of origin, with the majority of articles originating from the United States (10) and the Netherlands (5), followed by Sweden (4), United Kingdom/England (3), Canada (2), Denmark (1), and Taiwan (1). Twenty articles provided a clear conceptual and/or operational definition of frailty, of which 13 articles (52%) included quantitative objective or performance-based measures for frailty. Frailty definitions were sometimes difficult to locate and were embedded in the background or literature review; articulation of participant inclusion/exclusion criteria in the methods was sometimes unclear. Critical appraisal of the articles using CASP checklist identified 13 studies (52%) at risk for researcher bias due to insufficient information about participant recruitment; relationship between the researcher and the participant; reliance on subjective judgment of the researcher, a healthcare professional, or a family member to classify or identify frail participants; and lack of clear operational criteria for frailty (see Table 2, questions 4 and 5).

### 3.2. Older Age and Physical Impairment

Researchers cited older age and physical impairments as the primary frailty markers in the articles in this review. While all articles cited 65 years of age and older as the age cutoff, age greater than 80 years was a primary frailty indicator in nine articles (36%) [16, 17, 22, 23, 35, 38, 41]. In eight articles, frailty was subjectively determined by the researcher or a proxy such as a health professional or family member [18, 19, 22, 23, 35, 38, 39, 41]. For example, Becker [32] described frailty as the presence of chronic physical impairments in older individuals that the health professional would view as putting them at risk. Nicholson and colleagues [22, 23] determined frailty based on recommendations from an interdisciplinary care team that considered old age, inability to carry out activities of daily living, dependence, and vulnerability to physical decline. In several articles, dependence on caregivers and the healthcare system was a frailty indicator [16, 17, 19, 36, 41, 44]. A majority of articles in this review cited impairments in activities of daily living as inclusion criteria for frailty. However, most did not include information about assessment parameters and how measures for physical function were administered (self-reported or provider-administered) or scored [20, 21, 26, 27].

### 3.3. Operational Definition for Frailty and Objective Measures

About half of the articles employed objective or performance-based measures to characterize frailty in the participant sample (*n* = 13, 56%) (see Table 3). Four articles used validated screening tools for frailty such as grip strength, timed-up-and-go test, chair stands, and gait speed [3]; however, information about the tool and its scoring was absent. Measures for psychological function (e.g., cognition and mood) were included in seven articles, whereas three articles incorporated social factors such as living alone, social isolation, social support, and hours of care needed. Eight articles differentiated frail and nonfrail status [24, 25, 28, 29, 34, 36, 40, 45].

### 3.4. Synthesis of Conceptual Definitions of Frailty

**Figure 2:**
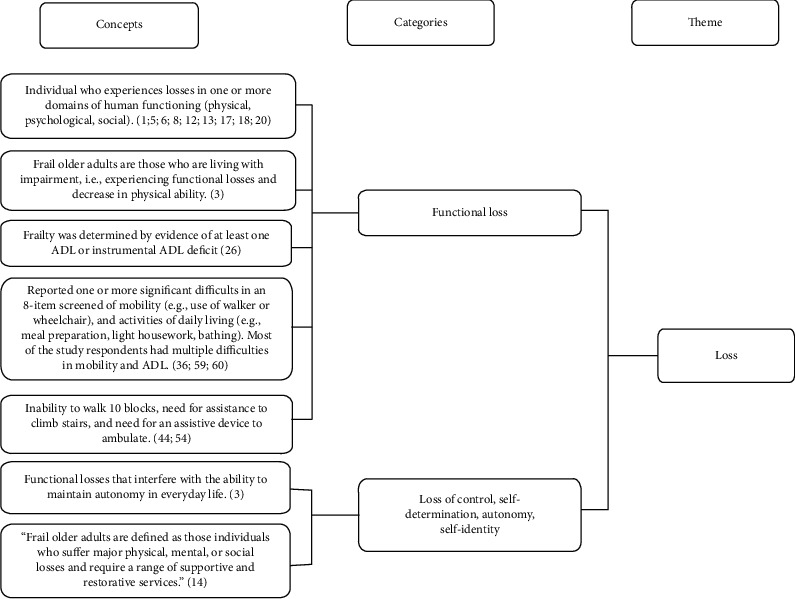
Data aggregation and classification of concepts into categories and themes.

**Figure 3:**
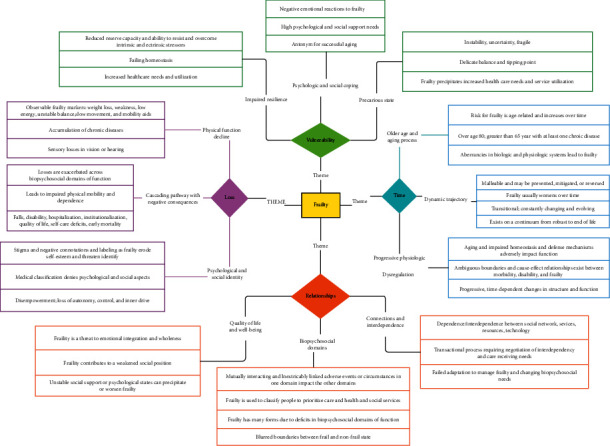
Concept map for frailty from the synthesis.

How researchers conceptually defined frailty in the qualitative research studies was examined using concept mapping. Synthesis of the 25 articles in this review yielded 10 categories and four themes: Time, Vulnerability, Loss, and Relationships. The themes, categories, and exemplar statements are summarized in Table 4.  Figure 2 provides an example of concept mapping and data reduction for one of the themes, and Figure 3 provides a graphical display of the concept map for the four themes.

#### 3.4.1. Theme 1: Time

Time was characterized by older age, aging process, dynamic trajectory, and progressive physiologic dysregulation. Frailty as an age-related and progressive condition was temporally described as an evolving trajectory with accumulation of health problems and impairments. Frailty was also characterized as a dynamic state that follows a continuum from robust to end-of-life. There is also a tipping point in the frailty trajectory in which the accumulated burden of disease and psychosocial challenges lead to transition from frailty risk to frailty as a reality. Transitional points on the frailty continuum signal opportunities to prevent, delay, or reverse frailty and accelerate its progression.

#### 3.4.2. Theme 2: Vulnerability

Vulnerability was portrayed as impaired resilience, a precarious state, and psychological and social coping. Frailty is highly unstable and unpredictable, with waning reserve, loss of resilience, and reduced capacity to resist stressors. Frailty is marked by increased risk for adverse outcomes, with differential risk appreciated in marginalized, minority, and immigrant populations and subgroups of older men and women. Frailty is a personal, subjective experience that could be triggered or worsened by negative emotions such as worry, sadness, fear, and anger. Psychological vulnerability arising from negative emotional experiences supersedes the physical experience and increases the risk for frailty. Social vulnerability was characterized by increasing dependency and insufficient social support and resources. Vulnerability associated with frailty requires coping strategies to counteract deterioration.

#### 3.4.3. Theme 3: Loss

Loss was reflected as a physical function decline, cascading pathway with negative consequences, and negative psychological and social identity. A central feature of frailty is the loss of capacity across biopsychosocial domains of function with increasing dependence. Loss-related markers of frailty included weakness, low energy, fatigue, weight loss, instability, vision and hearing deficits, impaired mobility, and need for mobility aids or human assistance. From a psychosocial perspective, frailty was a depersonalizing experience with loss of identity, control, autonomy, and self-determination. Frailty could also be triggered by losses due to bereavement and new functional impairment. Medical classification of frailty could be interpreted by individuals who frail as a threat to psychological well-being and loss of identity.

#### 3.4.4. Theme 4: Relationships

Finally, Relationships referred to biopsychosocial domains of function, quality of life and well-being, and connections and interdependence. Limitations of the dominant biomedical model that frames frailty in the empirical literature were acknowledged; frailty does not fit the medical model and is best represented as a complex system forming an integrated whole. There is a blurring of boundaries between frail and nonfrail states. Frailty threatens quality of life through compromised emotional integration, unstable social support, and weakened social position: frailty affects not only the individual, but also the social network and requires new connections to manage changing needs.

## 4. Discussion

### 4.1. Concordance in Conceptual Definitions of Frailty

In this qualitative systematic review of 25 articles, researchers conceptually defined frailty as a complex, multidimensional syndrome that evolves from underlying vulnerability, physiologic derangements, and loss of resilience manifested through dynamic interactions across the biopsychosocial domains of human function with greater risk for adverse outcomes. The four themes yielded in the synthesis identified concepts that align with the WHO Clinical Consortium on Healthy Aging Report [1] in which frailty is “…a clinically recognizable state in which the ability of older people to cope with every day or acute stressors is compromised by an increased vulnerability brought by age-associated declines in physiological reserve and function across multiple organ systems” (p. viii). Similarly, a concept analysis of frailty in the quantitative literature defined frailty as a tenuous state of health resulting from the complex interplay of physiological, psychosocial, and environmental stressors and is associated with numerous adverse health outcomes [46]. The conceptual definitions of frailty elaborated by researchers in the synthesis highlight human wholeness and the fact that psychological and social factors are as important as physical factors in frailty [47]. This evidence supports the growing body of evidence that articulates the relevance of a biopsychosocial model for frailty and provides a basis for moving away from organ- and disease-based approaches to frailty toward a more holistic, health- and wellness-based approach in geriatric care [1, 6, 7].

### 4.2. Methodological Deficits

A major finding is that many articles in this systematic review suffered from poor methodological quality. Lack of specificity in detailing how frailty was operationally defined raised questions as to whether the study population was frail. The quality appraisal using the CASP checklist determined over half of the articles (*n* = 13) could be considered biased and potentially ageist due to reliance on age and physical impairments as primary frailty criteria; there is strong evidence that all older adults who experience physical limitations are not frail [5]. Qualitative investigations can improve the description of a complex, real-world phenomenon such as frailty. However, if study participants are misjudged as frail, their reflections on frailty would be based on their own opinions and not personal, direct experience, which threatens the veracity of the findings.

To our knowledge, this qualitative systematic review provides the first synthesis of conceptual and operational definitions of frailty that were used for participant selection in qualitative research on the experience of living with frailty among frail community-living older adults. Recent similar efforts have been undertaken in the quantitative literature. Yaksic et al. [48] found that, out of 490 research study abstracts reviewed, only 348 (16%) had a complete definition of frailty that included the name of the frailty measure, the variables used in the measure, and the scoring for levels of frailty. In a systematic review of frailty definitions applied in 78 quantitative research studies, Junius-Walker et al. [46] found that many studies lacked clear frailty definitions and inclusion criteria and recommended five components that constituted a comprehensive definition of frailty. In agreement with our findings, these investigators endorsed concepts such as multiple dimensions of frailty, emphasis on a function focused, holistic approach, intrinsic vulnerability and capacities, and interacting environmental factors that influence frailty over a focus on the physical and biological aspects [49]. Sezgin et al. [50] conducted a systematic review and thematic analysis of frailty definitions in quantitative research and review articles (*N* = 86) and found an overemphasis on physical aspects of frailty with few studies addressing psychosocial domains; here, only three studies were cited from the qualitative literature. Taken together, this body of work, including the present systematic review, reinforces the importance of clearly defining and operationalizing frailty in qualitative research as this will facilitate accurate interpretation of study findings and facilitate the transferability of this knowledge in future research and clinical practice. Articulating specific inclusion criteria is important in qualitative research because it helps ensure that participants can provide the information necessary to address the research question and facilitates cross-study comparisons [51]. Deficits in participant sampling limits the number of qualitative research studies that would be eligible for inclusion in a qualitative metasynthesis, which is a mechanism to aggregate findings from qualitative research in order to yield new information [10].

### 4.3. Older Chronological Age as Synonymous with Frailty

Although research indicates that advancing age increases risk for frailty, chronologic age is only loosely correlated with biological age and is not the most reliable indicator for frailty [4]. In addition, although older age, comorbidity, and disability may overlap in the frailty experience, particularly in more advanced stages, they are distinctly different [52, 53]. Similarly, while advanced age brings a higher likelihood of multiple and interacting chronic diseases that may lead to frailty, it is notable that not all older people with comorbidity are frail, and younger persons may experience frailty [2, 54, 55]. Importantly, a hallmark of aging is the wide diversity of the aging experience; there is no typical older person [1]. Frailty is also found in middle-aged adults, especially minority groups such as African Americans where frailty not only develops earlier but also follows a more severe course due to health disparities and disadvantages in opportunities for developing healthy lifestyles and good health across the life course [1, 2]. Accelerated aging and frailty may be evidenced in individuals younger than 70 years due to health disparities arising from economic, educational, and health disadvantages in contrast to more robust 85-year-old individuals with lifelong access to resources and healthy lifestyles [56]. Thus, qualitative research that studies frailty only in older adults risks failing to detect frailty in younger, vulnerable populations who may experience different health circumstances and a steeper frailty trajectory [56].

### 4.4. Physical Impairment

The most common operational definition of frailty in this review focused primarily on physical function and limitations in activity of daily living. This finding contrasts with investigations and position papers that articulate distinctions between frailty and disability [5, 53]. An interdisciplinary consensus conference of international experts conferred that the most often used definition of physical frailty involved the evaluation of five physical-function-related domains (nutrition, energy, physical activity, mobility, strength) to identify older adults at high risk for adverse health outcomes; however, there was agreement that frailty is different from disability until its later stages [57]. Deficits in physical function often accompany comorbidities and may also be due to barriers in physical environments.

### 4.5. Holistic, Biopsychosocial Perspectives on Frailty

Despite the predominance of older age and physical impairments as key empirical indicators for frailty in this review, the conceptual definitions and descriptions of frailty in the qualitative research studies endorsed a more holistic perspective that recognizes the psychosocial domains in the scientific literature on frailty assessment [5, 6, 46, 51] although there is no agreement on which indicators [58–60]. Psychosocial factors such as depression, anxiety, quality of life, stressful life events, and resilience are recognized as correlates of frailty [47]. Cognitive frailty is characterized by the co-occurrence of physical frailty and cognitive impairment [61]. Social frailty is the accumulation of multiple social risk factors related to socioeconomic status, social support, social engagement, and social behaviors that can adversely impact health outcomes [62].

Notably, none of the articles in this review included spirituality as a factor in frailty, although research indicates that spirituality may be an important aspect of psychological health that moderates the negative effects of frailty [63]. Spirituality may be especially salient in certain population; for example, in a study using focus groups of African American men and women, spirituality was identified as a significant driver in the prevention and mitigation of frailty [64]. Qualitative research on frailty assessment that incorporates measures for psychosocial function and spirituality is needed, because frailty is about not only physical changes but also psychological, social, and spiritual factors that may precipitate and mediate such changes.

### 4.6. Empirical Indicators of Frailty

There is continued debate about the ideal frailty assessment that is applicable in research and clinical practice. In the scientific literature, frailty has been operationally defined in three major frameworks: (1) the phenotype for physical frailty quantified by objective criteria and performance-based measures (e.g., weight loss, weakness, exhaustion, slowness, and low physical activity) [53]; (2) according to a deficit accumulation framework represented by the proportion of a range of deficits (30–70) that are present, which reflects greater frailty [65, 66]; and (3) as a multidimensional biopsychosocial construct based on comprehensive geriatric assessment [7]. The present systematic review included articles dating back to 1994 since the search strategy extended to database inception. While this allowed for a broad representation of articles for the synthesis, methodologies for both qualitative research and frailty assessment have changed. More recent articles included empirical indicators and quantitative measures for frailty, such as handgrip strength, gait speed, and validated chair stand tests for frailty with demonstrated predictive properties [3]. Several articles in this review included tests for vision and hearing since there is evidence linking frailty and sensory deficits [67]. However, measures for pulmonary function such as spirometry and peak expiratory flow were used in two articles without justification for their relevance to frailty. Tests for cognitive function and mood were also used in some of the more contemporary articles. Increasingly, opinions about frailty in the scientific literature indicate that focusing exclusively on physical frailty hinders a full understanding of frailty and its impact on the individual [68].

### 4.7. Racial, Ethnic, and Cultural Diversity

Although the articles in this review displayed some cultural diversity by authorship and country of origin, cultural aspects of frailty were addressed in only two articles that included African American participants [20, 21]. These findings concur with recent systematic reviews of frailty that observe deficits in addressing cultural factors in definitions of frailty in the quantitative literature [49, 50]. A study that translated the Tilburg Frailty Indicator for use in the Jordanian population found that the psychometric properties were similar for physical frailty, but not for psychological or social frailty; thus, modification for cultural relevance was required [69]. For example, in Jordanian culture, close-knit families help compensate for the decline in aging and frailty; thus, to be consistent with Jordanian culture, items such as “Do you live alone?” and “Do you sometimes miss having people around you?” were revised as “Have you felt alone?” because older adults often live with family members [69]. In a study of older Taiwanese adults, feedback about a frailty assessment tool determined that they felt it was not effective in addressing quality of life [70]. Future research is needed to ensure that frailty measures used in qualitative research are culturally and socially relevant and sensitive to variations in life experience to more comprehensively assess the frailty experience.

### 4.8. Stigma Associated with Frailty

One interesting finding in this review was the extent to which researchers discussed negative connotations and stigma associated with frailty [18, 19, 21, 23, 35, 38, 39, 41] 2003. Stigma is the co-occurrence of labeling, stereotyping, separation, status loss, and discrimination and may result in feelings of shame, fear, guilt, suffering, depression, isolation, reluctance to seek treatment, and decreased self-esteem [71]. The language of frailty used to classify a person's health status is socially constructed based on prevailing norms and stereotypes and may have a detrimental impact on the individual due to stigma. In recent initiatives by Age UK and British Geriatrics Society [8] to address the public health impact of frailty, the “Fit for Frailty” campaign reported that the vocabulary of frailty was a barrier to involving older individuals in their care. While there is utility for the term frailty, there is a need to change how it is viewed and talked about by using different terminology [9]. Furthermore, negative emotions engendered by the term frailty that is used in participant recruitment may also hinder participation in research. Nonstigmatizing synonyms for frailty are needed to facilitate communication and recruitment in research contexts, since the term frailty may offend, frighten, or turn away potential participants. Several strategies are recommended for communicating with frail individuals that include avoidance of the term “frailty” and using language that promotes independence, enablement, and resilience [68, 72]. The concept of intrinsic capacity as posited by the World Health Organization is central to the prevention and mitigation of frailty; dialogue that frames frailty using perspectives of empowerment, capacity, and capability may foster resilience and resistance to frailty [1]. Public service messaging and health education should be tailored for subgroups based on gender, since the frailty experience among men and women differs [47]. Qualitative research can elaborate on these issues and uncover how resilience mediates frailty and identify terminology to incorporate a broader and more balanced understanding of frailty [68, 72].

The purpose of this review was not to discern or endorse a definition of frailty, but to represent how it is defined in qualitative research for participant selection inclusion criteria. Qualitative research including metasynthesis of qualitative research findings can inform understanding of a complex, ambiguous phenomenon such as frailty and contribute new knowledge about this condition from insights provided by the persons who are affected by it. Future research is needed in different age, racial, ethnic, and cultural groups to elucidate what is frailty, how a person becomes frail, what is its natural history, how is it managed, and what can be done to prevent it. To achieve these objectives, qualitative research should clearly articulate the conceptual and operational definition of frailty, the inclusion and exclusion criteria, and how the data will be analyzed to differentiate the voices of participants who are frail or nonfrail. Our findings signal a call to action for the use of a standardized approach to reporting how frailty is defined in abstracts and studies as proposed by Yaksic et al. [48] which will facilitate qualitative metasynthesis studies to capture the increasing volume of qualitative research and facilitate knowledge transfer and accurate reporting of scientific work in frailty that is needed to catalyze initiatives to improve healthcare.

### 4.9. Strengths and Limitations

This qualitative systematic review included qualitative research from multiple geographic regions and professional disciplines. A majority of articles were from European, Asian, and American countries with a lack of representation of global geographic regions such as Africa, low- and middle-income countries, and some of the most populous countries such as China, India, and Russia. There were contributions from diverse disciplines; however, in the era of globalization and team science, future research enterprises should adopt transdisciplinary collaborations to advance frailty science. While this review involved a comprehensive search of multiple databases, potentially relevant articles may have been missed, and there may be an unintended bias toward articles published in the peer-reviewed English language literature. The quality of the included articles varied, but we retained all relevant articles for a more enriched synthesis. We employed a systematic, rigorous method for data extraction, content analysis, and concept mapping for the synthesis; however, the possibility of overlooking key information or misinterpretation during these processes exists. Finally, this review concentrated on definitions of frailty in qualitative research focused on studies in community-living frail older adults; how researchers define frailty in qualitative research in other contexts such as acute care hospitals and long-term care nursing homes may differ and merits further inquiry.

## 5. Conclusion

Frailty is a compelling global public health issue that significantly impacts individuals, families, communities, and society. The anticipated increase in the incidence and prevalence of frailty and its adverse consequences underscores the need to better understand frailty. The findings from this systematic review of the qualitative literature on definitions of frailty in qualitative research on the lived experience of frailty among frail older adults indicate that frailty was conceptually defined as a multidimensional, biopsychosocial, holistic construct but was often operationally defined by older age and functional impairment. Over half of the studies were appraised to be at risk for researcher bias due to lack of clear criteria to operationalize frailty in the study methodology. This review underscores the need for clear articulation of frailty defining characteristics and objective indicators in study abstracts and methods in future qualitative research. Such transparency will facilitate cross-study comparisons and development of qualitative metasynthesis and meta-analysis studies which are necessary to expedite the development of the science base that is necessary to drive future research and guide improvements in the care for frail older adults.

## Figures and Tables

**Table 1:** Description of the qualitative research articles in the study sample (*N* = 25).

	First author, date; 1^st^ author discipline; country of origin; [ID]	Aim/purpose	Conceptual description or definition of frailty	Operational definition of frailty	Sample *N*; age mean (range) in years; gender; race
1	Andreasen et al., 2015; occupational therapy; Denmark; [34]	To validate the Tilburg Frailty Indicator on content by exploring the experience of daily life of community-dwelling frail elderly shortly after discharge from an acute admission, in relation to the physical, psychological, and social domains of the TFI.	A dynamic state affecting an individual who experiences losses in one or more domains of human functioning (physical, psychological, social) that are caused by the influence of a range of variables, which increases the risk of adverse outcomes and negatively impacts well-being.	Tilburg Frailty Indicator, a 15-item self-administered questionnaire: physical domain (8 items), psychological domain (4 items), social domain (3 items). Frailty cutoff score accounts for five of the 15 frailty indicators.	*N* = 14; 80.6 (69–93); 7 men, 7 women; Caucasian

2	Becker, 1994; social science; USA; [35]	To explore the meanings older persons attach to autonomy and decreases in physical abilities associated with frailty.	Chronically dependent older people, those who are living with a variety of physical and/or cognitive impairments and experiencing functional losses and decrease in physical ability that interfere with ability to maintain autonomy in everyday life.	80 years of age and older; frailty determined by opinion of health professional including presence of chronic impairments that health professionals would view as putting people at risk.	*N* = 19; ≥80; 12 men, 16 women; Hispanic and Caucasian, 2 African American women

3	Claassens et al., 2014; medicine; Netherlands; [36]	To investigate the concept of healthcare-related perceived control from the viewpoint of frail older adults >65 years.	Frail older adults cope with multiple and/or chronic health conditions that likely require more extensive forms of healthcare.	Frail determined by scoring below cutoff scores, on at least two of the six following domains: BMI <23; cognitive function (MMSE <24); vision and hearing acuity; grip strength (handheld dynamometers); physical activity (how often & how long they walked, cycled, performed household activities, played sports) during 2 past weeks.	*N* = 32; 80.5 (65–96); 13 men, 19 women; Caucasian

4	Donlan, 2011; social work; USA; [37]	To identify how frail Mexican American elders socially constructed the meaning of community-based care they received.	Not described; frailty and disability often accompany old age, especially among marginalized immigrant populations.	Age ≥65 years and having a disability. Requiring assistance with ADLs.	*N* = 6; 77.5 (66–89); 3 men, 3 women; Hispanic

5	Ebrahimi et al., 2012; nursing; Sweden; [16]	To discover and reveal the meaning of experienced health through the analysis of frail elders' descriptions.	As people age, their reserve capacity decreases, and the risk of morbidity and frailty increases; a multidimensional geriatric syndrome of disability; vulnerability and reduced capacity.	Age ≥80 years or ≥65 years with one or more chronic diseases; those who depended on help in at least one ADL and sought emergency treatment in a hospital.	*N* = 22; 79.5 (67–92); 11 men, 11 women; Caucasian

6	Ebrahimi et al., 2013; nursing; Sweden; [17]	To explore and identify what influences frail older adults' subjective experiences of good health.	A biological geriatric syndrome of reductions in physiological reserve capacity and impairment of defense mechanisms against stress and disease which implies a risk of multimorbidity and dependence on others.	Age ≥80 years or ≥65 years with one or more chronic diseases; those who depended on help in at least one ADL and sought emergency treatment in a hospital. Frailty determined by a count.	*N* = 22; 79.5 (67–92); 11 men, 10 women; Caucasian

7	Ekelund et al., 2014; occupational therapy; Sweden; [38]	To explore community-living frail older persons' conceptions of self-determination.	A physiological state of increased vulnerability to stressors that result from decreased physiological reserve; related to risk for disability and comorbidity; the presence of various diseases, age discrimination, and paternalism impact frailty; dependency is an important aspect of frailty.	Age ≥80 years or ≥65 years with one or more chronic diseases; those who depended on help in at least one ADL and sought emergency treatment in a hospital.	*N* = 15; 80.5 (68–92); 8 men, 7 women; Caucasian

8	Evans et al., 2001; radiology; USA; [39]	To investigate whether frail older women with a positive perception of health would desire to take a more active role in their healthcare.	Frail older adults are defined as those individuals who suffer major physical, mental, or social losses and require a range of supportive and restorative services.	Frailty criteria/measures not clearly specified, but older women were “…categorized by age as frail older adults.” Convenience sample of frail older women who were scheduled for ultrasound examination and whose health status indicates ability to participate in interviews.	*N* = 4; 82.25 (76–90); 4 women; not reported

9	Grenier & Hanley, 2007; social work; Canada; [19]	To explore the life experiences of frailty.	Definition derived from the social label of frailty and not physical function and was related to the presence of comorbidities. The social context of frailty as the “little old lady” of small stature, being fragile and weak, is associated with assumptions that shape the gendered experience of older women. Frailty framed in context of resistance to dominant notions of aging and gender, to challenge social constructs and expectations for aging and frailty. Frailty is also a term used by health professionals to assess a person's need for public services to meet physical needs.	Frailty criteria/measures not specified. Frailty determination is based on clinical judgment and home care eligibility by health professionals for half of the sample, and the other half were active in an advocacy organization but fell outside the classification because they did not receive public services due to lack of physical need, interest, or financial resources to pay privately.	*N* = 12; >55; 12 women; not reported

10	Grenier, 2006; social work; Canada; [18]	To explore the distinction within older women's narratives which represent a clash between the professional construct of frailty and the lived experiences of older women.	Frailty is contextually and socially located; one aspect of the person's appearance (i.e., of “being frail”) comes to stand for the total identity. “Being” frail is related to the imposition of a classification that is medical and functional in nature; there are emotional aspects of frailty that lie within the experiences of impairment, disability, and decline in later life that may contradict the medical and social nature of frailty. “Feeling” frail may or may not correspond with experiences of impairment or disability. Certain events may trigger frailty: new impairment, loss, bereavement, evolving chronic illness.	Diverse older women in sources of inequalities, e.g., ability, age, race, ethnicity, culture, and socioeconomic status; six were considered frail based on clinical judgment and home care eligibility and six women were classified as not frail because they did not receive public services due to lack of physical need, interest, or financial resources to pay privately.	*N* = 12; not reported; 12 women; “diverse”

11	Hammar et al., 2014; neuroscience; Sweden; [40]	To explore experiences of self-determination when developing dependence in daily activities among community-dwelling persons 80 years and older.	Frailty is a continuum of 3 phases: robust prefrail, fully frail; a dynamic concept; directly related to decreased ability to perform daily activities independently.	80 years and older; frailty based on eight frailty indicators: weakness, fatigue, weight loss, physical activity, poor balance, slow gait speed, visual impairment, and cognition; classified as nonfrail (0 indicators), prefrail (1–2 indicators), and frail (3 or more indicators).	*N* = 11; 87 (84–95); 5 men, 6 women; Caucasian

12	Jett, 2002; nursing; USA; [20]	To explore the process of help-seeking and help giving by older rural African Americans and how certain of the most vulnerable and least known elders seek help for day-to-day needs.	Frailty not defined; survival of frail elders and role of ADLs and IADLs for day-to-day functioning described; frail elders are most vulnerable with the least known needs and at greater risk for losses and unmet needs which can be mitigated with help-seeking behaviors.	Age ≥65 years, living alone, and evidence of at least one ADL (range 6 [complete independence] to 36 [complete dependence]) or IADL deficit (range 8 [complete independence] to 24 [complete dependence]), and “knowledgeable about aging and frailty.”	*N* = 41, 9 frail; not reported; 9 women; African American

13	Jett, 2003; nursing; USA; [21]	To examine the meaning of aging from the perspective of older African American women living in rural areas.	Frailty not defined; the study focused on ethnography of the aging, fragility, and survival of rural elderly African Americans and learning who is identified as aged, how aging is defined and culturally determined, and what it means to be old.	Age ≥65 years, living alone, and frail based on at least one ADL or IADL deficit; ADL score: 6 (complete independence) to 36 (complete dependence), and IADL score 8 (complete independence) to 24 (complete dependence).	*N* = 9; 84 (77–94); 9 women; African American

14	Kaufman, 1994; nursing; USA; [41]	To investigate ways in which frailty is defined, framed, and understood by older persons, their family members, and healthcare providers in the context of a multidisciplinary geriatric assessment service; to explore the process of increasing of frailty in advanced old age, how they attempt to understand, accept, manage, and combat frailty within the context of the American healthcare system and the mechanisms employed to cope with and solve the variety of problems it creates.	Frailty increases with advancing age; a dynamic adaptational process that is open to multiple interpretations. The medicalization of frailty overshadows psychological, emotional, and behavioral aspects of aging and frailty. Frailty is socially produced in response to powerful discourses in American culture. Frailty is proposed when someone conceives there to be a lived problem with a very old person; either the old person has a condition that is worsening or spreading to other body systems or areas of the person's life, or family members can no longer cope with caring for the person and focus on symptoms or behaviors as problems.	Age ≥80 years; receiving geriatric assessment services; and perceived by family members, friends, or health professionals to be at risk with a change in condition, health decline, and need for medical care, social support, and/or supervision so that they could remain in the community.	*N* = 3; ≥80; 3 women; not reported

15	Kuo et al., 2012; nursing; Taiwan; [42]	To cross-examine results between perception of frailty and physical assessment outcomes then try to establish frailty indicators for elderly people in Taiwan.	Frailty indicates a dynamic model and a balance of psychological and physical strength to counterthreats to health; a decline in physical reserve capacity and ability to resist stress.	65 years and older; Barthel index for ADL, IADL, grip strength (handheld dynamometer), timed-up-and-go test, paper folding test, spirometry, vision test, incontinence, body mass index, waist-hip ratio, body fat composition, Mini-Mental State Exam, Geriatric Depression Scale.	*N* = 10; 69.5 (65–74); 10 women; Asian

16	Moss et al., 2007; sociology/anthropology; USA; [43]	To learn the meanings and themes that underlie attitudes of frail old men who live in the community and behaviors in relation to food and eating.	Not described.	Frailty based on eight-item screener of mobility (e.g., use of walker or wheelchair) and activities of daily living (e.g., meal preparation, light housework, and bathing).	*N* = 21; 83 (76–95); 11 men, 10 women; 12 Caucasian, 3 African American

17	Nicholson et al., 2012; nursing; United Kingdom; [22]	To understand the experience of home-dwelling older people living with frailty over time in order to develop the empirical evidence base for this group and to consider more fully how narratives of frailty can shape person-centered care provision.	Frailty is an antonym for successful aging and a synonym for the increasing infirmities that accompany aging and the slow dwindling dying trajectory of many elders. This trajectory is gradual and unpredictable, encompassing accumulated and multiple health problems, which at some point tips the person into the dying phase. The social construction of the fourth age as a loss of agency and bodily self-control is linked to frailty.	Frail persons were defined by the interdisciplinary care team based on advancing age, unable to carry out IADLs and considered to be vulnerable to physical decline.	*N* = 17; 94 (86–102); 5 men, 12 women; Caucasian
18	Nicholson et al., 2013; nursing; United Kingdom; [23]	To understand the experience over time of home-dwelling older people deemed frail, in order to enhance the evidence base for person-centered approaches to frail elder care.	Frailty describes the condition of people vulnerable to adverse health outcomes in later life and includes a broader definition that includes social functioning, social relationships, and psychological frailty, e.g., anxiety and loneliness encompassing social, psychological, and physical domains.	Frail elders were purposively selected by the multidisciplinary care team (community nurses, speech therapist, physiotherapists, occupational therapists, care support workers, geriatricians) based on age ≥85 year, unable to carry out IADLs and considered to be vulnerable to physical decline.	*N* = 15; 94 (86–102); 5 men, 10 women; Caucasian

19	Niesten et al., 2012; dental science; Netherlands; [25]	To identify and examine how natural teeth contribute to the quality of life of dentulous people who are elderly and frail and how frailty influences the impact of having natural teeth on quality of life.	Frailty is a state of reduced psychological or physical reserve in combination with an increased risk for adverse outcomes such as falls, disability, and institutionalization. Frailty impacts health in general and the value that people ascribe to their oral health and their subjective dental care needs and demands.	Age ≥65 years and frailty score based on eight domains: social coping, psychosocial function, personal care, mobility, motor function, medical care, behavior disorders, and care needs per week. Score ranged from 0 to 10, where score of “1” indicated mild frailty and “6” severe frailty; persons scoring 7–10 were excluded. Scoring was determined by a medical authority.	*N* = 38; 79.9 (65–97); 11 men, 27 women; 2 Indonesian women, 25 Caucasian women, and 11 Caucasian men.

20	Niesten et al., 2013; dental science; Netherlands; [24]	To explain how frailty influences dental service use and oral self-care by older people.	A state of reduced psychological or physical reserve in combination with an increased risk for adverse outcomes such as falls, disability, and institutionalization; a dynamic state affecting an individual who experiences losses in one or more domains of human functioning (physical, psychological, social) which likely negatively affects dental service use and oral hygiene-related behaviors.	Age ≥65 years and a frailty score based on eight domains: Social coping, psychosocial function, personal care, mobility, motor function, medical care, behavior disorders, and care needs per week. Score ranges from 0 to 10, where scores of “1” indicated mild frailty and “6” severe frailty; persons scoring 7–10 excluded. Scoring determined by a medical authority.	*N* = 51; 24 being 65–80, 27 being ≥80 years; 16 men, 35 women; not reported

21	O'Connor, 1994; social work; England; [44]	To recognize the affective reality of elderly persons' experiences in the life review of frail elderly people who are living alone.	Frail elderly people who are living alone, housebound, and/or in need of assistance with basic activities of daily living and/or have emotional and/or social problems (which may include perceived inability to care for themselves) are in a socially vulnerable position.	Randomly selected homebound social work clients who need ADL assistance. Frailty markers to describe the sample: falling in the past year, having partial or total loss of use of an arm or a leg, prone to heart attacks and/or acute attacks of bronchitis or asthma, unable to get out of bed, walk indoors or outside, climb stairs, and/or bathe.	*N* = 134; ≥65; 28 men, 114 women; not reported

22	Porter, 1999; nursing; USA; [26]	To explore a neglected realm of frail older women's experience of falling to the floor and trying to get up while at home alone.	Not defined, but it was stated that frail older persons are at risk for falls and participants had physical function deficits that were indicators of frailty.	Women aged 80 years and older, living alone at home, self-rated health of less than excellent, history of a fall. Frailty determined by three criteria: inability to walk 10 blocks, need for assistance to climb stairs, and need for assistive device to walk.	*N* = 18; 89.5 (83–96); all women; not reported

23	Puts et al., 2009; nursing; Netherlands; [28]	To describe the meaning that older community-dwelling persons attach to frailty.	Frailty is often used to describe a state in which older persons are, in a delicate balance, at risk for many adverse outcomes such as falls, disability, institutionalization, and death, which may have a negative effect on quality of life.	Frailty determined by eight frailty markers: low body mass index, low peak expiratory flow, poor vision and hearing ability, incontinence, low sense of mastery, depressive symptoms, and physical inactivity. Frailty defined as having three or more markers and nonfrail defined as no frailty markers.	*N* = 25; 78.7 (67–90); 14 men, 11 women; Caucasian

24	Puts et al., 2007; nursing; Netherlands; [29]	To describe the meaning of quality of life from the perspective of frail and nonfrail older community-dwelling persons in Netherlands.	A state in which older persons are in a delicate balance and at risk for many adverse outcomes such as falls, disability, institutionalization, and death.	Frailty determined by eight frailty markers: low body mass index, low peak expiratory flow, poor vision and hearing ability, incontinence, low sense of mastery, depressive symptoms, and physical inactivity. Frailty defined as having three or more markers and nonfrail defined as no frailty markers.	*N* = 25; 78.7 (67–90); 14 men, 11 women; Caucasian

25	Schoenborn et al., 2018; medicine; USA; [45]	To examine perceptions and informational needs about frailty among older adults.	A medical syndrome consisting of specific physical symptoms, leading to multiple adverse outcomes including falls, hospitalization, functional dependence, and death.	Age 65 years and older. Frailty based on Fried frailty criteria: weakness (handgrip strength), exhaustion, weight loss, physical activity, gait speed, and cognition (Mini-Mental State Exam); classified as nonfrail (0 indicators), prefrail (1–2 indicators), or frail (3 or more indicators).	*N* = 29; 76.3 (>65); 8 men, 21 women; Caucasian (21), African American (7), other (1)

**Table 2:** Quality appraisal: CASP Qualitative Studies Checklist and evaluative criteria.

Criteria	1	2	3	4	5	6	7	8	9	10	11	12	13	14	15	16	17	18	19	20	21	22	23	24	25
1. Was there a clear statement of the aims of the research? Considering the goal of the research, why it is thought it is important, relevance.	Y	Y	Y	Y	Y	Y	Y	Y	Y	Y	Y	Y	Y	Y	Y	Y	Y	Y	Y	Y	Y	Y	Y	Y	Y
2. Is a qualitative methodology appropriate? If the research seeks to interpret or illuminate the actions and/or subjective experiences of research participants.	Y	Y	Y	Y	Y	Y	Y	Y	Y	Y	Y	Y	Y	Y	Y	Y	Y	Y	Y	Y	Y	Y	Y	Y	Y
3. Was the research design appropriate to address the aims of the research? Did the researcher justify the research design?	Y	Y	Y	Y	Y	Y	Y	N	C	C	Y	Y	Y	Y	Y	N	C	C	Y	Y	Y	C	Y	Y	Y
4. Was the recruitment strategy appropriate to the aims of the research? Researcher explained how the participants were selected, why the participants selected were the most appropriate to provide the type of knowledge sought by the study, and why some people chose not to participate.	Y	C	Y	Y	Y	Y	C	N	N	N	Y	N	N	N	Y	N	N	N	Y	Y	N	N	Y	Y	Y
5. Has the relationship between researcher and participants been adequately considered? Has the researcher critically examined their own role, potential bias, and influence during (a) formulation of the research questions and (b) data collection, including sample recruitment and choice of location.	Y	N	Y	Y	Y	Y	N	N	N	N	Y	N	N	N	Y	N	N	N	Y	Y	N	N	Y	Y	Y
6. Have ethical issues been taken into consideration? Approval has been sought from the ethics committee	Y	Y	Y	Y	Y	Y	Y	Y	C	C	Y	Y	Y	Y	Y	Y	Y	Y	Y	Y	Y	Y	Y	Y	Y

Rating: Y: yes; N: no; C: cannot answer. CASP: Critical Appraisal Skills Programme, https://casp-uk.b-cdn.net/wp-%20content/uploads/2020/10/CASP_RCT_Checklist_PDF_Fillable_Form.pdf.

**Table 3:** Quantitative measurement of frailty in qualitative research studies (*N* = 13).

Author, year; frailty cut point	Physical performance tests	Cognition function	Mood-self-report	Metrics	Self-report questionnaire
Andreasen et al., 2015; frailty = 5/15					TFI: physical domain (feeling healthy, weight loss, vision, hearing, walking, balance, hand strength, tiredness), psychological domain (memory, mood, anxiety, coping), social domain (living alone, social isolation, social support)

Claassens et al., 2014; frailty = 2/6	Grip strength, vision, hearing	MMSE		BMI	Physical activity

Hammar et al., 2014; frailty = 3/8 indicators	Grip strength, gait speed, balance, vision	MMSE			Endurance/physical activity, fatigue, weight loss

Jett, 2002; 2003 frailty cut point = one ADL deficit					ADL and IADL, measurement not specified

Kuo et al., 2012; frailty cut point not specified	Barthel index for ADL, IADL, grip strength, timed up-and-go test, vision, hearing, paper folding test, spirometry	MMSE	GDS	BMI, waist-hip ratio, body fat composition	Incontinence

Niesten et al., 2012, 2013; slight, moderate, severe frailty based on level of care needed		Behavior disorders, psychosocial function			Motor function, mobility, personal care needed, medical care needed, hours of care needed per week; social coping

Porter et al., 1999, 2001; measurement not specified					Inability to walk 10 blocks, need for assistance to climb stairs, need for assistive device to walk

Puts et al., 2007, 2009; frailty = 3/8 indicators	Peak expiratory flow		Depression, CES-D	BMI	Physical activity, vision, hearing, incontinence, sense of mastery

Schoenborn et al., 2018; frailty = 3/5 indicators	Hand grip strength, gait speed	MMSE			Physical activity, weight loss, exhaustion, health literacy, numeracy, self-reported health status

Note: ADL: activities of daily living (controlling bowel and bladder, grooming, toileting, feeding, transferring, walking, bathing, climbing stairs, and dressing); IADL: instrumental activities of daily living (telephone use, meal prep, money & medication management, light & heavy housekeeping, shopping, and local travel); BMI: body mass index; CES-D: Center for Epidemiologic Studies Depression Scale; MMSE: Mini-Mental State Exam; GDS: Geriatric Depression Scale; TFI: Tilburg Frailty Indicator.

**Table 4:** Frailty concept map themes, categories, and statements.

Frailty		
Theme	Category	Sample statements
Time	Older age and aging process	Risk for frailty is age-related and increases over time
		Over age 80; greater than 65 years; “old-old”; fourth age
		Aberrancies in biologic and physiologic systems lead to frailty
		Gradual and unpredictable in the aging process
	Progressive physiologic dysregulation	Aging and impaired homeostasis and defense mechanisms adversely impact function
		Ambiguous boundaries and cause-effect relationships exist between morbidity, disability, and frailty
		Progressive, time-dependent changes in structure and function
	Dynamic trajectory	Malleable and may be prevented, mitigated, or reversed
		Frailty usually worsens over time; slow dwindling dying trajectory
		Transitional; constantly changing and evolving
		Exists on a continuum from robust to end-of-life

Relationships	Biopsychosocial domains of function	Domains are interrelated; mutually interacting and inextricably linked; adverse events in one domain impact the other domains
		Frailty is used to classify people to prioritize care and health and social services
		Frailty has many forms due to deficits in biopsychosocial domains of function
		Blurred boundaries between frail and nonfrail state; distinctions between feeling frail and being frail
	Quality of life and well-being	Frailty is a threat to emotional integration and wholeness
		Frailty is associated with social isolation, weakened social position
		Unstable social support or psychological states can precipitate or worsen frailty
	Connections and interdependence	Dependence/interdependence between social network, services, resources, technology
		Transactional process requiring negotiation of interdependency and care receiving needs
		Failed adaptation to manage frailty and changing biopsychosocial needs

Loss	Physical function decline	Observable frailty markers: weight loss, weakness, low energy, unstable balance, slow movement, and mobility aids
		Accumulation of chronic diseases
		Sensory losses in vision or hearing
	Cascading pathway with negative consequences	Losses are exacerbated across biopsychosocial domains of function
		Leads to impaired physical mobility and dependence
		Falls, disability, hospitalization, institutionalization, quality of life, self-care deficits, early mortality
	Psychological and social identity	Stigma and negative connotations and labeling as frailty erode self-esteem and threaten identity
		Medical classification denies psychological and social aspects
		Disempowerment; loss of autonomy, control, and inner drive

Vulnerability	Impaired resilience	Reduced reserve capacity and ability to resist and overcome intrinsic and extrinsic stressors
		Failing homeostasis and risk for adverse outcomes
		Increased healthcare needs and utilization
	Precarious state	Instability, uncertainty, fragility
		Delicate balance and tipping point; fragility
		Frailty precipitates increased healthcare needs and service utilization
	Psychologic and social coping	Negative emotional reactions to frailty; diminished autonomy
		High psychological and social support needs; negatively impacted by social isolation, living alone
		Antonym for successful aging

## Data Availability

No data were used to support this study.
